# Papilledema Secondary to Barotrauma in a Young Adult With Severe Status Asthmaticus With Ventilatory Failure, Pneumothorax, and a Complex Clinical Course

**DOI:** 10.7759/cureus.50044

**Published:** 2023-12-06

**Authors:** Iyad Y Idries, Vasilii Khristoforov, Ruchi Yadav, Avtar Sur, Vivek Yadav, Ahmed Hossny, Junior Kalambay, Mohammad Zaman

**Affiliations:** 1 Internal Medicine, Brookdale University Hospital Medical Center, Brooklyn, USA; 2 Intensive Care Unit, Brookdale University Hospital Medical Center, Brooklyn, USA; 3 Hematology and Oncology, Brookdale University Hospital Medical Center, Brooklyn, USA; 4 Pulmonary and Critical Care, State University of New York Downstate Health Sciences University, New York, USA; 5 Internal Medicine, Danylo Halytsky Lviv National Medical University, Lviv, UKR; 6 Internal Medicine, West Side Medical Center, Metuchen, USA; 7 Critical Care Medicine, Brookdale University Hospital Medical Center, Brooklyn, USA

**Keywords:** intracranial pressure, mechanical ventilation, pneumothorax, barotrauma, papilledema

## Abstract

Intubation and mechanical ventilation are common therapeutic interventions in intensive care unit settings. Barotrauma is a known complication of using positive pressures in a tissue defined by extra alveolar air in locations where it is not generally found in patients receiving mechanical ventilation. Several clinical manifestations of barotrauma include pneumothorax, subcutaneous emphysema, pneumoperitoneum, pneumomediastinum or pneumopericardium, air embolization, and hyperinflated left lower lobe. However, papilledema is an unreported and uncommon complication we observed in one of our patients, making it a unique presentation. We present the case of a young male patient intubated for asthma exacerbation requiring mechanical ventilation with subsequent development of papilledema. Our case report highlights the importance of knowing this rare complication of barotrauma as early commencement of lung-protective strategies will help prevent it.

## Introduction

Asthma exacerbations are well-recognized for their varied and unpredictable presentations. This intriguing case highlights the atypical complications that can arise from severe asthma and underscores the importance of vigilant post-intubation monitoring to detect rare conditions such as papilledema [1].

## Case presentation

A 23-year-old male with a medical history of asthma and neonatal heart surgery presented to the emergency department (ED) complaining of sudden-onset respiratory distress, vomiting, and pleuritic chest pain. He reported that these symptoms had developed one day before admission, initially beginning with throat discomfort and progressing to shortness of breath, a mild cough productive of clear and sometimes yellow sputum, and pleuritic chest pain. Remarkably, the patient denied any fever, abdominal pain, or diarrhea. His typical use of an albuterol inhaler at home, averaging around 10 times per week, had provided no relief. Notably, he disclosed a history of nocturnal inhaler use approximately five times weekly, with a baseline peak expiratory flow rate of approximately 200. Furthermore, he had no recent sick contacts or travel history and denied any history of smoking, alcohol use, or illicit drug abuse but admitted to using marijuana.

Upon initial evaluation in the ED, the patient presented as hemodynamically stable and afebrile. Laboratory findings of blood gases are presented in Table 1. The patient’s complete blood count showed a white blood cell count of 8.8 and a negative troponin.

**Table 1:** Initial arterial blood gas and repeat blood blood gas analysis three hours after admission.

Component	Initial arterial blood gas	Repeat arterial blood gas
pH, arterial 7.35–7.45	7.16	7.17
pCO_2_, arterial 35.0–45.0 mmHg	64.2	67.0
pO_2_, arterial 80.0–110.0 mmHg	123.0	106.0
HCO_3_, arterial 22.0–26.0 mmol/L	22.7	24.5
O_2_ saturation, arterial 95.0–100.0%	98.8	98.2
Lactate, arterial 0.7–2.1 mmol/L	3.2	3.4
Base excess, arterial -3.0–3.0 mmol/L	-7.3	-5.6
Total hemoglobin, arterial 12.9–16.7 g/dL	15.1	15.9
Methemoglobin 0.0–1.5%	0.4	0.3
Sodium, whole blood 133.0–145.0 mmol/L	143	141
Potassium, whole blood 3.7–5.3 mmol/L	4.5	3.8
Chloride, whole blood 95–100 mmol/L	111	109
Glucose, blood 70.0–99.0 mg/dL	157	157
Normal Aa gradient mmHg	8.25	8.25

The COVID-19 test returned negative. A chest X-ray exhibited hyperinflated lungs without infiltrates. The patient received two doses of IV magnesium sulfate (1 mg), IV solumedrol (125 mg), terbutaline, IM epinephrine, and albuterol nebulization. Regrettably, despite these aggressive interventions, the patient’s respiratory status deteriorated, prompting his transfer to the intensive care unit (ICU) for further management.

Intensive care unit course

**Figure 1:**
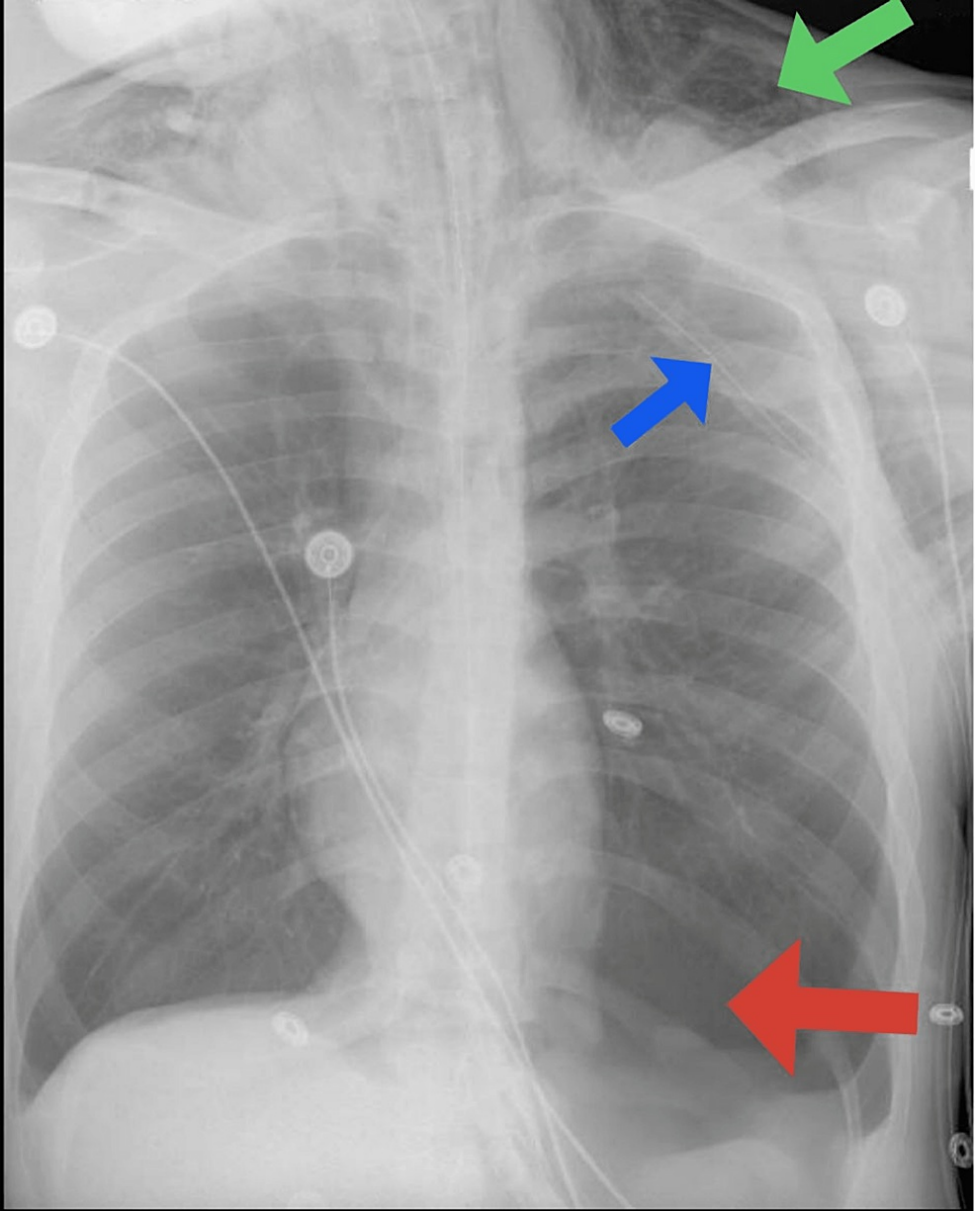
Left basilar pneumothorax (red arrow) and supraclavicular subcutaneous emphysema (green arrow) with a chest tube placed in the left chest (blue arrow).

Upon arrival in the ICU unit, the patient became increasingly agitated, and the severity of his wheezing escalated, along with attempts to remove the bilevel positive airway pressure mask. Consequently, the decision was made to secure his airway through intubation. Central venous and arterial lines were placed, and he was initiated on phenylephrine to manage his hemodynamic status. Left-sided pneumothorax was seen post-intubation, likely secondary to barotrauma and a chest tube was inserted (Figure 1). The patient’s ventilator settings were meticulously adjusted based on serial arterial blood gases (ABGs) to optimize his respiratory parameters. Serial ABGs demonstrated gradual improvement, as seen in Table 2.

**Table 2:** Repeat blood gas analysis.

Component	Repeat arterial blood gas
pH, arterial 7.35–7.45	7.46
pCO_2_, arterial 35.0–45.0 mmHg	46.1
pO_2_, arterial 80.0–110.0 mmHg	141
HCO_3_, arterial 22.0–26.0 mmol/L	32.4
O_2_ saturation, arterial 95.0–100.0%	99.4
Lactate, arterial 0.7–2.1 mmol/L	0.6
Base excess, arterial -3.0–3.0 mmol/L	7.3
Total hemoglobin, arterial 12.9–16.7 g/dL	13.6
Methemoglobin 0.0–1.5%	0.6
Sodium, whole blood 133.0–145.0 mmol/L	143
Potassium, whole blood 3.7–5.3 mmol/L	4.6
Chloride, whole blood 95–100 mmol/L	105
Glucose, blood 70.0–99.0 mg/dL	118
Normal Aa gradient mmHg	8.25

During this complex ICU course, the patient had to be paralyzed with cisatracurium and sedated with midazolam drip for lung-protective ventilation, and the patient developed subcutaneous emphysema. Eventually, he was successfully weaned off pressors and paralytic medications, transitioning to ketamine and ampicillin/sulbactam for ongoing care. Serial chest X-rays unveiled the presence of free air under the diaphragm, a finding attributed to a traumatic chest tube placement during cardiothoracic surgery placement of the chest tube. No immediate surgical intervention was required, postulating that the air had translocated from the thoracic cavity into the subdiaphragmatic space, which was noted post-extubation. The patient was briefly re-intubated for airway protection, after which he was cautiously weaned from sedation. He successfully passed a spontaneous breathing trial and was extubated, and his chest tube was removed.

Floor course

Following the challenging ICU course, the patient was transferred to a medical floor. During his time on the floor, he remained comfortable on room air, and no wheezing was appreciated during auscultation. On consultation, pulmonology recommended outpatient respiratory allergy panel testing and pulmonary function testing to further evaluate the patient’s respiratory status. However, the patient’s prolonged stay in the ICU had left him deconditioned, resulting in an unsteady gait. Consequently, rehabilitation services were initiated, with physical therapy to improve his mobility and overall physical condition.

Surprisingly, the patient reported experiencing blurry vision in his left eye, accompanied by subconjunctival hemorrhage. Concerned about potential intracranial pathology, ophthalmology was consulted. The evaluation raised suspicion of an aneurysm or brain tumor due to papilledema. The ophthalmological examination showed a dilated left pupil with a relative afferent pupillary defect, bilateral papilledema OS > OD, macula striations OS, and subconjunctival hemorrhage in the left eye. A CT of the orbit and a computed tomography angiogram of the head were performed to rule out these potentially life-threatening conditions. Thankfully, both imaging studies returned negative for any intracranial pathology, excluding an aneurysm or tumor. This led to the conclusion that the patient’s papilledema likely resulted from barotrauma sustained due to positive pressure ventilation.

## Discussion

The trajectory of the 23-year-old male patient’s clinical presentation gravitates compellingly toward the unexpected diagnosis of papilledema amid primary respiratory concerns. Papilledema, an optic disc swelling usually resulting from elevated intracranial pressure (ICP), emerged as a singular anomaly requiring astute evaluation.

Traditionally, papilledema is concomitant with severe pathologies that block cerebrospinal fluid (CSF) outflow channels and rarely conditions that increase CSF production [2]. Barotrauma is a feared complication of mechanical ventilation defined as high-pressure-induced injury [3]. The incidence of barotrauma is estimated to be approximately 2.9% in mechanically ventilated patients [4].

Barotrauma can have several clinical and radiological manifestations, including pneumothorax, pulmonary interstitial emphysema, subcutaneous emphysema, pneumoperitoneum, pneumo-retroperitoneum, lung cysts, and pneumopericardium [5]. However, papilledema is not commonly observed in this context, and our comprehensive diagnostic assessment ruled out other potential causes. A compelling concept has surfaced, proposing that the increased central venous pressure (CVP), sinus venous pressure, and subsequent elevation of ICP resulting in observed papilledema may be attributed to barotrauma caused by intubation, positive pressure ventilation, and pneumothorax [6].

**Figure 2:**
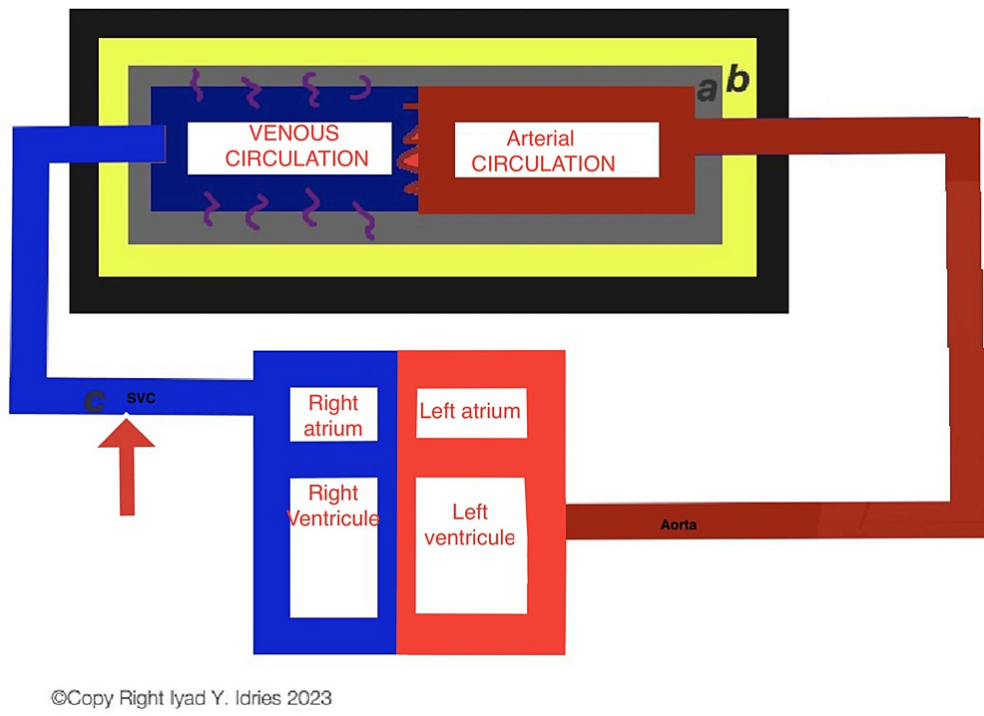
Visual representation of the anatomical representation of the pathophysiology of barotrauma. a: CSF. b: Brain. c: Site of pathology in barotrauma (red arrow). The figure describes the site of the pathology and how it can lead to backflow into the superior vena cava, eventually increasing the hydrostatic pressure inside the venous circulation of the brain which subsequently increases the intracranial pressure leading to papilledema.

According to the Davson equation, resistance to venous outflow leads to the elevation of ICP [7]. The elevation of intrathoracic pressure resulting from pneumothorax and positive pressure ventilation has been observed to raise CVP (Figure 2). It may contribute to elevated ICP, believed to be the underlying cause of barotrauma-induced papilledema. In a typical scenario, the autoregulation of ICP is a preventive mechanism against its elevation. The relative compliance of the lung and chest wall affects the transmission of airway pressure to pleural pressure [8]. However, in specific situations where lung compliance is diminished and positive end-expiratory pressure (PEEP) is administered, there is the possibility of ICP escalation [9]. This theory underscores physiological systems’ pervasive interconnectivity and therapeutic interventions’ potential wide-reaching implications. Patients with acute severe asthma develop serious neurological sequelae due to critically raised ICP before or during mechanical ventilation [10].

**Figure 3:**
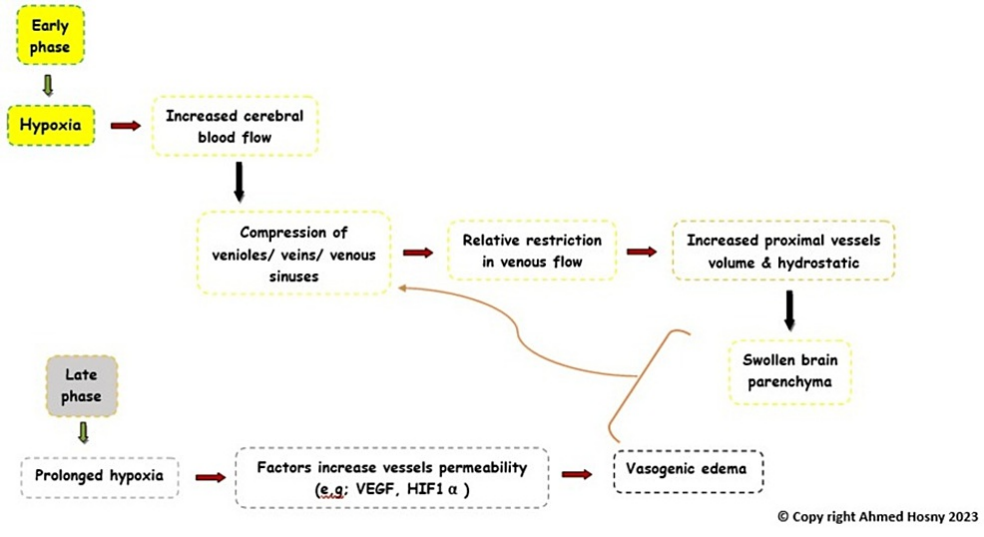
Suggested mechanism of hypoxia leading to brain edema and increased intracranial pressure.

Other pathophysiological processes include hypoxia, leading to decreased cerebral blood flow, leading to compression of venioles, veins, and venous sinus, which increases the hydrostatic pressure within the proximal blood vessels, leading to parenchymal edema [1] (Figure 3).

In essence, although the primary focus of the patient was on respiratory discomfort, the unexpected presence of papilledema revealed intricate interactions within the pathophysiology of the human body. This instance underscores the necessity of a comprehensive approach, particularly when confronted with secondary but noteworthy indications such as papilledema. This statement is a strong reminder to healthcare professionals to maintain a high level of awareness, recognizing that even prevailing clinical accounts can be connected to peripheral yet equally important physiological occurrences.

This case and those previously presented emphasize that patients with acute severe asthma, who developed profoundly hypercarbia lasting for more than 24 hours, with initial hypoxia in addition to increased intrathoracic pressure in the setting of barotrauma, may have critically raised ICP before or during mechanical ventilation leading to potentially serious neurological sequelae. Recognition of this risk is critical to optimizing adjunctive therapy.

## Conclusions

This case report highlights the challenges in managing severe asthma exacerbations, focusing on the uncommon complication of papilledema caused by elevated CVP and hypoxia during intubation and ventilation. It emphasizes the importance of interdisciplinary teamwork, precise management, and diligent follow-up in complex medical cases. The report underscores the need for healthcare professionals to remain open to diverse clinical presentations and complications, even when following established protocols. Additionally, it discusses the impact of PEEP on ICP and cerebral oxygenation in severe asthma, emphasizing the importance of considering volume status and respiratory mechanics to mitigate adverse effects.
